# Non-Alcoholic Fatty Liver Disease and Extra-Hepatic Cancers

**DOI:** 10.3390/ijms17050717

**Published:** 2016-05-12

**Authors:** Claudia Sanna, Chiara Rosso, Milena Marietti, Elisabetta Bugianesi

**Affiliations:** Division of Gastroenterology, Department of Medical Sciences, A.O. Città della Salute e della Scienza di Torino, University of Turin, 10126 Turin, Italy; sanna.cla@gmail.com (C.S.); chiara.rosso84@tiscali.it (C.R.); milena.marietti@gmail.com (M.M.)

**Keywords:** fatty liver, colorectal cancer, adipokines, gut microbiota

## Abstract

Non-alcoholic fatty liver disease (NAFLD) is a leading cause of chronic liver disease but the second cause of death among NAFLD patients are attributed to malignancies at both gastrointestinal (liver, colon, esophagus, stomach, and pancreas) and extra-intestinal sites (kidney in men, and breast in women). Obesity and related metabolic abnormalities are associated with increased incidence or mortality for a number of cancers. NAFLD has an intertwined relationship with metabolic syndrome and significantly contributes to the risk of hepatocellular carcinoma (HCC), but recent evidence have fuelled concerns that NAFLD may be a new, and added, risk factor for extra-hepatic cancers, particularly in the gastrointestinal tract. In this review we critically appraise key studies on NAFLD-associated extra-hepatic cancers and speculate on how NAFLD may influence carcinogenesis at these sites.

## 1. Introduction

Nonalcoholic fatty liver disease (NAFLD) is one of the most common causes of chronic liver disease worldwide, with an estimated global prevalence of 25% in adults and around 10% in children [1,2,3]. The term NAFLD includes two distinct conditions with different histologic features and prognoses: non-alcoholic fatty liver (NAFL) and non-alcoholic steatohepatitis (NASH) [4]; the presence of steatohepatitis and significant fibrosis are considered harbingers of adverse outcomes in individuals with NAFLD and are associated with an increased risk for morbidity and mortality through hepatic and non-hepatic complications [5,6,7]. In descending order, the majority of deaths in patients with NAFLD are, first, attributed to cardiovascular events, and, second, to malignancies at both gastrointestinal (liver, colon, esophagus, stomach, and pancreas) and extra-intestinal site (kidney in men, and breast in women), while end-stage liver disease is the third cause of death [8,9]. NAFLD is traditionally considered the hepatic manifestation of metabolic syndrome (MetS) and an impressive body of evidence indicates an increased general risk of cancer in subjects with MetS, particularly in the gastrointestinal tract. In this setting, NAFLD can either share common risk factors (*i.e.*, obesity and type 2 diabetes) or actively mediate some pathogenic mechanism, as in the case of liver cancer (hepatocellular carcinoma, HCC). Excluding the latter one, colorectal cancer (CRC) has been consistently associated with NAFLD thus far [10,11]. The mechanisms underlying the link between NAFLD and risk of neoplasms are not fully elucidated but they probably stem from the bidirectional relationship between NAFLD and MetS [12,13,14]. In this review we critically appraise the key studies on the association between NAFLD and extra-hepatic cancers and speculate on how NAFLD may influence carcinogenesis at these sites.

## 2. Nonalcoholic Fatty Liver Disease (NAFLD) and Colorectal Cancer

The association between NAFLD and CRC is the best investigated in literature (details are summarized in Table 1). Almost all of the studies showed a higher prevalence of colorectal lesions in patients with NAFLD compared to patients without. Hwang and colleagues presented the first evidence for an association of NAFLD with an increased rate of colorectal adenomatous polyps [15]. In their study, a population of 2917 participants was investigated via colonoscopy, abdominal ultrasonography, and liver tests. The prevalence of NAFLD was 41.5% in the adenomatous polyp group *versus* 30.2% in the control group; with multivariate analysis, NAFLD was associated with a three-fold increased risk of colorectal adenomas. This preliminary finding was confirmed in a large retrospective cohort study of 5.517 Korean women, where a two-fold increase in the occurrence of adenomatous polyps and a three-fold increase in the risk of colorectal cancer was found in patients with NAFLD compared to controls. However, the presence of NAFLD had no influence on the prognosis of colorectal cancer and, in particular, on the disease recurrence during follow-up [16]. Among NAFLD patients, those with histological diagnosis of NASH harbinger the most increased risk for CRC. In a cross-sectional study patients with NAFLD, diagnosed by both proton magnetic resonance spectroscopy and liver biopsy, had a significantly higher rate of colorectal adenomas (34.7% *vs.* 21.5%) and advanced neoplasms (18.6% *vs.* 5.5%) than healthy controls [17]. Almost half of NAFLD patients with advanced neoplasm had right-sided colorectal carcinoma. Importantly, CRC was more often found in patients with NASH compared to those with simple steatosis (51.0% *vs.* 25.6% and 34.7% *vs.* 14.0%). NASH remained associated with a higher risk of both adenomas (Odds Ratio (OR) 4.89) and advanced neoplasms (OR 5.34) even after adjusting for demographic and metabolic risk factor, thus, the authors concluded that screening colonoscopy should be strongly recommended in these patients [17]. In the largest study performed so far in Europe, male patients with NAFLD had significantly more colorectal adenomas and early colorectal cancers compared to those without NAFLD [18]. Multivariate regression analysis confirmed an independent association of colorectal adenomas with NAFLD (OR 1.47) [17]. Data stemming from cross-sectional studies have also been replicated longitudinally. In a prospective study where 1522 subjects underwent paired colonoscopies, while the index colonoscopy was negative in all of them, the incidence of *de novo* adenoma development was increased by 45% in those with NAFLD [19]. Lastly, a Danish cohort study evaluating the global risk of cancer in hospitalized patient showed an increased risk of CRC in those with fatty liver compared to the general population, but no difference was noticed between alcoholic and non-alcoholic fatty liver [20].

In contrast, only two studies failed to demonstrate an increased incidence of colorectal adenomas in patients with NAFLD compared to healthy controls [21,22]. The first one found a higher burden of adenomas in patients with NAFLD, but data did not reach a statistical significance, probably for the smaller sample size and the younger median age. The second one remarkably showed a lower prevalence of CRC in NAFLD patients but a higher risk for CRC in the presence of insulin resistance; however it is well known that both raised alanine aminotransferase (ALT) levels and ultrasound can underestimate the diagnosis of NAFLD.

Overall, it appears that NAFLD patients are more likely to have multiple polyps [23], more often localized more in the right and transverse segments of colon [17,23]; importantly, patients with histologic diagnosis of NASH are at higher risk for adenomatous polyps with high grade dysplasia (HGD) compared to those with simple fatty liver [17]. The relationship between NAFLD and CRC once again emphasizes the importance of a healthy lifestyle to prevent and treat the MetS and its systemic manifestations. Certainly these data suggest that NAFLD patients should undergo a closer surveillance for CRC risk according to screening guidelines [24]. If the evidence of this association will be further confirmed in larger population studies, probably these patients should be screened in advance and total colonoscopy considered as the preferred screening method, as neoplasms are more commonly found in the proximal colon [19,24].

## 3. NAFLD and Cancers in Other Sites

The association of NAFLD with other extra-hepatic cancers is less proven. In the previously-mentioned Danish study all-cancers risk was increased by 70% in subjects with fatty liver, either alcoholic or non-alcoholic [20]; however, those with NAFLD had a higher risk of pancreatic and kidney cancer (standardized incidence ratio (SIR) 3; 95% confidence interval (CI) 1.3–5.8 and SIR 2.7; 95% CI 1.1–5.6, respectively), malignant melanoma (SIR 2.4; 95% CI 0.8–5.6) and cancer metastases from primary unspecified sites (SIR 6.3; 95% CI 1.3–18.4), while those with alcoholic fatty liver had a higher risk for lung and breast cancer (SIR 2.2; 95% CI 1.7–2.8 and SIR 1.5; 95% CI 0.9–2.2, respectively). The latter observation contrasts with another study where a higher prevalence of breast cancers was observed in patients with ultrasound diagnosed NAFLD compared with healthy controls (63% *vs.* 48%, respectively) [26]. The burden of data available is currently too limited to draw definite conclusions about a specific role of NAFLD, as the link can be mediated by visceral obesity, which in turn is strongly associated to fatty liver in the so-called “central-axis” of obesity. A recent review summarized the well-recognized role of visceral obesity in the onset and development of various cancers [27], including CRC [28,29,30,31], esophageal [32,33,34,35,36,37,38] and pancreatic cancer [39], breast [40], thyroid [41], and probably prostate cancer [42]. What is currently unknown is whether both NAFLD and visceral obesity are just markers of an increased risk of cancers or also active players in this process. With this caveat in mind, we will briefly examine the association between NAFLD, visceral obesity, and cancers other than CRC.

### 3.1. Esophageal and Gastric Cancer

Esophageal cancer is the eighth most common form of cancer worldwide, and the World Cancer Research Fund has identified obesity as a major risk factor, able to increase the risk up to four-fold compared with lean populations [43]. Several more recent studies suggest a stronger impact of visceral fat distribution rather than body mass index (BMI) *per se* [37,44,45], but no study specifically examined hepatic fat. Strikingly, the association between visceral obesity and esophageal adenocarcinoma is independent of gastro-oesophageal reflux disease (GORD), and possibly mediated by adipose tissue insulin resistance and chronic inflammation [32,46,47]. A possible direct link between NAFLD and gastric cancer has been suggested in a recent study, performed on 1840 patients undergoing upper endoscopies over a six-month time frame; despite the limited number of gastric cancer diagnosed, the prevalence of NAFLD in subjects with gastric cancers was higher compared to the average in the Turkish population [48].

### 3.2. Pancreatic Cancer

In 2007 the World Cancer Research Fund/American Institute for Cancer Research (WCRF) definitively established the association between pancreatic cancer and overweight/obesity. A meta-analysis published in 2012 showed a linear increase between pancreatic cancer risk and waist circumference, with a relative risk (RR) of 1.11 (95% CI 1.05–1.18) for every 10 cm increase, and waist-to-hip ratio, with a RR of 1.19 (95% CI 1.09–1.31) for every 0.1 unit increment [39]. In a meta-analysis performed in 2012, MetS has been identified itself as a neoplastic risk factor, with a RR of 1.58 (*p* < 0.0001) for pancreatic cancer in female gender, possibly mediated by decreased physical activity, consumption of high-calorie dense foods, high dietary fat intake, low fiber intake, and oxidative stress [49]. As for esophageal cancer, NAFLD can be implicated in this association, although no direct evidence is yet available.

### 3.3. Renal Cancer

In addition to smoking and dietary habits, whose association with renal cancer is well established, some of the components of MetS, such as obesity and hypertension, have been recognized etiological factors and listed in specialist guidelines [50,51]. In a large study of seven European cohorts, high level of a metabolic risk score, based on the combination of BMI, blood pressure, and plasma levels of glucose, total cholesterol and triglycerides, was linearly and positively associated to higher incidence of renal cell cancer (risk increase per standard deviation of metabolic risk score increment: 43% in men and 40% in women) [52]. In patients with cT1a renal cell carcinoma visceral fat, assessed by computed tomography (CT) scan, is strongly associated with Fuhrman grade, the most frequently used neoplastic nuclear grading system for kidney, and is an independent predictor of high-grade renal cell carcinoma (RCC) [53]. In a study performed on 118 consecutive patients undergoing surgical treatment for RCC, adiponectin levels are inversely proportional to the severity of disease, with the lower levels in patients with metastatic cancer [54].

### 3.4. Breast Cancer

The association between breast cancer risk in postmenopausal women and components of MetS has been provided by several large studies [49,55,56,57]. In combined analyses of two case-control study on 3869 postmenopausal women with breast cancer and 4082 postmenopausal control cases, authors registered a higher neoplastic risk in women with MetS than those without (OR 1.75; 95% CI 1.37–2.22). In the analysis of distribution of cases and controls according to individual components of the syndrome, the resulting corresponding odds ratios were 1.33 (95% CI 1.09–1.62) for diabetes, 1.19 (95% CI 1.07–1.33) for hypertension, 1.08 (95% CI 0.95–1.22) for hyperlipidemia, 1.26 (95% CI 1.11–1.44) for BMI ≥ 30 kg/m^2^, and 1.22 (95% CI 1.09–1.36) for waist circumference ≥88 cm [56]. In a study on 2092 patients, surgically treated for stage I–III invasive breast cancer in the previous five years and followed-up over 2.8 years on average, MetS appeared a major determinant of the occurrence of additional related events, such as specific mortality, presence of distant metastasis, or local recurrences and incidence of contralateral breast cancer [58]. Although each component was associated with an increased risk of cancer recurrence, the risk associated with the full syndrome was the highest, likely to be the expression of a general dysmetabolic condition rather than of a specific trait.

### 3.5. Prostate Cancer

The link between dysmetabolic factors, NAFLD and prostate cancer is controversial. In a systematic review and meta-regression analysis, including 31 cohort and 25 case-control, for every five kg/m^2^ increment in BMI, authors described a 1.05 relative risk (95% CI 1.01–1.08), higher in patients with progressed diseases than localized diseases [59]. Two studies specifically investigated the role of NALFD. In the first one, NAFLD was found to be protective against neoplastic recurrence after radical prostatectomy for prostate cancer in 293 consecutive patients [60]. The NAFLD group showed significantly longer time-to-recurrence compared with patients without NAFLD both in the training and validation set (hazard ratio: 0.33 and 0.22; 95% CI 0.16–0.69, and 95% CI 0.11–0.43, respectively). The second one analyzed the development of malignancies and the specific site of disease in 1600 US-defined NAFLD subjects and in 1600 matched hepatitis C virus (HCV)-infected patients: prostate cancer developed in 12.6% of NAFLD compared to 3.5% in HCV patients [61], and the incidence of prostate cancer in NAFLD was higher than in the general population.

## 4. Putative Role of Insulin Resistance and Gut Microbiota in the Development of Extra-Hepatic Cancers in NAFLD

Although the most extensive evidence of a possible mechanistic link between NAFLD and extra-hepatic carcinogenesis currently comes from data on the pro-inflammatory and pro-carcinogenic effects of insulin resistance (IR), gut microbiota has been recently identified as a novel and intriguing player in the development of obesity, NAFLD and several types of cancer (details are summarized in Table 2). Patients with NAFLD are characterized by dysbiosis [62] and the liver stays at the cross-road of the complex interaction between changes in microbiota composition, IR, inflammation, and carcinogenesis [63,64]. Dysbiosis has been found in patients with colon cancer [65] and the possible correlation has been widely studied. Quantitative and qualitative alterations of gut microbiota lead to increased intestinal permeability through several mechanisms, including the regulation of tight junctions, such as zonulin-1, and occluding by toll like receptor 2 (TLR2) in the ileum. These alterations favor the translocation of bacterial metabolites and activation of TLRs via the recognition of microorganism-associated molecular patterns (MAMPs) and can promote tumorigenesis through the reduced release of the inflammasome-derived interleukin 18 (IL-18) and the increased IL-6 signaling which, in turn, protects normal and premalignant cells from apoptosis [11,66,67].

It is well known that host diet significantly impacts on gut microbial composition. Diet-induced NAFLD may be mediated by the myeloid differentiation factor 88 (MyD88)-dependent pathway [68]. This factor is an adaptor molecule, essential for the signaling through TLRs. It is recruited after the interaction among the microorganism-associated molecular patterns (MAMPs) and TLRs (particularly TLR4) and promotes the transcription of several pro-inflammatory cytokines through the activation of NF-κB or c-Jun NH_2_-terminal kinase (JNK) leading to the induction of IR. Loss-of-function mutation or knockout mice in TLR4 prevents IR induced by obesity underlying the important role of this receptor in the modulation of the innate immune system.

NAFLD and visceral adipose tissue are the main components of the axis of central obesity. In this setting, low-grade chronic inflammation and insulin resistance (IR) create a microenvironment suitable for cancer development through the stimulation of the insulin growth factor-1 (IGF-1) axis by hyperinsulinemia [9,69,70,71]. Through its proliferative and anti-apoptotic effects, this pathway can boost mutations favoring carcinogenesis [72,73]. Elevated serum levels of IGF-1 have been associated with prostate [74,75], colorectal [76], lung [77], and breast cancer [78]. Importantly, the insulin/IGF system is able to influence the risk of Barrett’s esophagus and of esophageal adenocarcinoma [37,79,80], although there is no full agreement about this [81].

Several adipokines, involved in the modulation of metabolism, inflammation and fibrogenesis, can also be involved in carcinogenic processes. Adiponectin has anti-carcinogenic effects mediated by its ability to stop colon cancer cell growth through the AMPc-activated protein kinase (AMPK) and to induce a caspase-dependent pathway resulting in endothelial cell apoptosis. Adiponectin can also directly inhibit tumor necrosis factor α (TNF-α), involved in tumor cell proliferation and angiogenesis. Since NAFLD patients have reduced serum levels of adiponectin, the above described mechanisms represent an interesting link between NAFLD and cancer development at both gastrointestinal and extra-intestinal site.

The pro-carcinogenic effects of leptin, especially in the presence of low adiponectin levels, have been widely investigated. In obese animal models, leptin acts as a growth factor for CRC at early stages through the activation of signal transducer and activator of transcription 3 (STAT 3) pathway [82]. In human colon cancer cells leptin is able to promote motility and invasiveness by activation of mitogen-activated protein kinase (MAPK) pathway [83]. A case-cohort study in post-menopausal women with CRC demonstrated that high plasma concentrations of leptin were associated with an increased risk for CRC [84]. In obese subjects the combination of high leptin and low adiponectin levels may also increase the risk of Barrett’s esophagus [85,86,87,88,89,90] and esophageal adenocarcinoma by enhanced cell proliferation and reduced apoptosis via extracellular signal-regulated kinase (ERK), p38 MAPK, phosphatidylinositol 3′-kinase/Akt, and Janus kinase-2 (JAK2)-dependent activation of cyclooxygenase-2 (COX-2) and prostaglandin E2 (PGE2). The association between leptin serum levels and the size of breast tumors has been summarized in a recent review [91]; higher leptin levels are related to a more aggressive disease, presence of metastasis and a lower survival rate [92] mostly in obese patients [93].

Finally, resistin can also be linked to obesity-related malignancies via activation of nuclear factor-κ B (NF-κB) pathway and amplification of the procarcinogenic effects of interleukin (IL)-1, IL-6 and TNF-α [94]. To date, a putative role of resistin has been suggested in breast cancer [94], non-small cell lung cancer [95] and in gastrointestinal tumors [96].

The low-grade chronic inflammation associated with IR also favors macrophages recruitment and massive release of several proinflammatory cytokines, such as IL-6 and TNF-α, into the systemic circulation. IL-6 induces the Janus kinase/signal transducer and activator of transcription (JAK/STAT) and MAPK pathways, stimulating cell proliferation and tumor progression, while TNF-α influences cancer angiogenesis, metastasis development and cell survival, growth, and differentiation [97,98,99]. Animal models have shown a relationship between TNF-α and several malignancies [100,101,102] including colorectal cancer [103]. Obese mice have higher TNF-α levels in the colonic mucosa, leading to β-catenin stabilization and increased transcription of the downstream Wnt pathway gene c-Myc [104]. IL-6 has been linked to renal cell carcinoma [105], gastric cancer [106], and colorectal cancer [107,108], through its modulation of several genes involved in proliferation, survival, and angiogenesis [109].

In consideration of the above described mechanisms, the increased risk of gastrointestinal cancers associated to NAFLD does not appear causal, although more extensive studies are required to demonstrate a direct link between NAFLD and cancers at various sites.

## 5. Conclusions

NAFLD is a complex multifactorial disease closely interrelated with obesity and type 2 diabetes, and shares with them a significant increased risk of several types of cancer. Beyond the risk of HCC, clearly mediated by NASH, substantial evidence is accumulating for a role of NAFLD as independent risk factor for cancers, particularly in the gastrointestinal tract. Once again, these preliminary, but intriguing, data convey that NAFLD patients require a multidisciplinary evaluation with a particular attention to the development of extra-hepatic complications. Further studies are necessary to better define high-risk NAFLD patients and effective screening strategies, but we encourage health care providers taking care of NAFLD patients to be vigilant for any signs and symptoms of cancer, particularly colorectal, and refer the patients for further assessment and management.

## Figures and Tables

**Table 1:** Principal studies on the association between nonalcoholic fatty liver disease (NAFLD) and colorectal neoplasms *.

Study	Country	Type of Study	Population Enrolled	Exclusion Criteria	NAFLD Diagnosis	Prevalence of Colorectal Lesions in Patients with NAFLD *vs.* Patients without NAFLD
Bhatt BD *et al.* [23] (2015)	USA	Retrospective	591 pts who completed LT evaluation (68 NAFLD *vs.* 523 non-NAFLD)	<50 years old at LT; IBD; history of multiple/recurrent adenomas; family history of CRC; known cancer-predisposing gene alteration; history of solid organ transplant; HIV pts; personal history of cancer	Biopsy + clinical criteria	Polyps prevalence: 59% *vs.* 40%; *p* < 0.003. OR (Odds Ratio) 2.16; *p* = 0.003 Adenomatous polyps prevalence: 32% *vs.* 21%; *p* = 0.04. OR 1.95, *p* = 0.02
Basyigit S *et al.* [22] (2015)	Turkey	Prospective observational	127 consecutive pts who underwent colonoscopy	Other causes of hepatic disease; incomplete colonoscopy; IBD; active gastrointestinal bleeding; history of colorectal surgery; history of CRC; hereditary cancer syndrome	US	Adenomas prevalence: 20% *vs.* 25.8%. OR 1 CRC prevalence: 4.6% *vs.* 24.2%. OR 1
Lin XF *et al*. [25] (2014)	China	Retrospective and consecutive cohort study	2315 community subjects who underwent a routine colonoscopy (263 NAFLD *vs.* 2052 non-NAFLD)	History of CRC, adenoma and polyp; history of other extraintestinal malignancies; contraindications to colonoscopy	US	Total colorectal lesions prevalence: 90.0% *vs.* 93.3% Adenomatous polyps prevalence: 44.5% *vs.* 55.7% CRC prevalence: 29.3% *vs.* 18%; *p* = 0.001. OR 1.868; 95% CI 1.360–2.567; *p* < 0.05
Wong VW-S *et al*. [17] (2012)	China	Cross-sectional	380 community pts + consecutive pts with biopsy proven NAFLD (in total 199 NAFLD *vs.* 181-non-NAFLD)	Other causes of hepatic disease; history of CRC or polyps; IBD; bowel symptoms including per rectal bleeding and altered bowel habit; prior CRC screening; contraindications to colonoscopy	Proton-magnetic resonance spectroscopy or liver biopsy	Total polyps prevalence: 52.8% *vs.* 38.7%; *p* = 0.057 Adenomatous polyps prevalence: 34.7% *vs.* 21.5%; *p* = 0.043. OR 1.61; 95% CI 0.9–2.9; *p* = 0.11 Villous polyps prevalence: 6% *vs.* 0.6%; *p* = 0.042 High grade dysplasia polyps prevalence: 18.1% *vs.* 5%; *p* = 0.002 Advance neoplasm prevalence: 18.6% *vs.* 5.5%; *p* = 0.005. OR 3.04; 95% CI 1.29–7.2; *p* = 0.011 CRC 1% *vs.* 0.6%; *p* = 0.65
Stadlmayr A *et al.* [18] (2011)	Austria	Cross-sectional	1211 consecutive pts who underwent screening colonoscopy (632 NAFLD *vs.* 597 non-NAFLD)	Incomplete colonoscopy; recent colorectal polypectomy, asymptomatic IBD; extraintestinal malignancies	US + exclusion of other causes of hepatic disease	Total colorectal lesions prevalence: 34% *vs.* 21.7%; *p* < 0.001 Tubular adenoma prevalence in men: 34.6% *vs.* 23.7%; *p* = 0.006 Rectum adenoma prevalence in men: 11% *vs.* 3%; *p* = 0.004 CRC prevalence in men: 1.6% *vs.* 0.4%; *p* < 0.001
Lee YI *et al*. [16] (2011)	South Korea	Retrospective cohort study	5517 women who underwent life insurance company health examinations (831 NAFLD *vs.* 4686 non-NAFLD)	Other causes of hepatic disease; history of receiving previous medical insurance benefits	US + exclusion of other causes of hepatic disease	Adenomatous polyps incidence: 628 *vs.* 185.2/10^5^ person year. RR 1.94; 95% CI 1.11–3.40 CRC incidence: 233.6 *vs.* 27/10^5^ person year. RR 3.08; 95% CI 1.02–9.34
Touzin NT *et al.* [21] (2011)	USA	Retrospective cohort study	233 patients who underwent screening colonoscopies (94 NAFD *vs.* 139 non-NAFLD)	Not available	US + liver biopsy	Adenomas prevalence: 24.4% *vs.* 25.1%; *p* = 1
Huang KW *et al*. [19] (2012)	Taiwan	Retrospective cohort study	1522 pts with two consecutive colonoscopies (216 with colorectal adenoma *vs.* 1306 without colorectal adenoma after negative baseline colonoscopy)	History of colorectal adenoma or CRC; adenomas during baseline colonoscopy; incomplete medical record data; alcohol consumption >20 g/day	US + exclusion of other causes of hepatic disease	NAFLD prevalence: 55.6% *vs.* 38.8%; *p* < 0.05. OR = 1.45; 95% CI 1.07–1.98; *p* = 0.016
Hwang ST *et al*. [15] (2009)	South Korea	Cross-sectional	2917 pts who underwent routine colonoscopy (556 with polyps *vs.* 2361 without polyps)	Incomplete colonoscopies; history of polypectomy; IBD; history of cancer; cancer detected during the study; pts with anticoagulant therapy; other causes of hepatic disease	US	NAFLD prevalence: 41.5% *vs.* 30.2%; *p* < 0.001. OR, 1.30; 95% CI 1.02–1.66; *p* = 0.034

* CI, confidence interval; CRC, colorectal cancer; IBD, intestinal bowel disease; LT, liver transplant; NAFLD, non-alcoholic fatty liver disease; OR, odds ratio; pts, patients; RR: relative risk; US, ultrasound.

**Table 2:** Putative mechanisms linking NAFLD and extra-hepatic cancers.

Mechanism	Effects	Extra-Hepatic Site
Insulin resistance		
↑ IGF-1 axis	Proliferative and anti-apoptotic effects	Prostate/colorectal/lung/Breast cancers, Barrett’s esophagus, esophageal adenocarcinoma
Dysfunctional adipose tissue		
↓ adiponectin/caspase activation ↓ adiponectin/TNF-α ↑ leptin/MAPK ↑ resistin/NF-κB	Anti-apoptotic effects Proliferation and angiogenesis Invasiveness, motility, lamellipodia formation	Gastrointestinal and extra-intestinal cancer Gastrointestinal and extra-intestinal cancer Colon/breast cancer, Barrett’s esophagus, esophageal adenocarcinoma Breast/gastrointestinal and non-small cell lung cancers
Inflammation		
IL-6/JAK/STAT3 and IL-6/MAPK TNF-α/Wnt/β-catenin	Proliferation Angiogenesis, differentiation and metastasis development	Renal/gastric/colorectal cancers Colorectal cancer
Gut microbiota		
MAMPs/TLRs Inflammasome-derived IL-18	Inflammation Anti-apoptotic effects	Colon cancer Colon cancer

IGF-1, insulin growth factor-1; IL, interleukin; MAMPs, microorganism-associated molecular patterns; MAPK, mitogen-activated protein kinase; NF-κB, nuclear factor-κ B; STAT3, signal transducer and activator of transcription 3; TLRs, toll-like receptors; TNF-α, tumor necrosis factor-α.

## Abbreviations

AMPK	AMPc-activated protein kinase
CI	confidence interval
COX-2	cyclooxygenase 2
CRC	colorectal cancer
ERK	extracellular signal-regulated kinase
HCC	hepatocellular carcinoma
HGD	high grade dysplasia
IBD	inflammatory bowel disease
IGF	insulin growth factors
IL	interleukin
IR	insulin resistance
LT	liver transplant
MAMPs	microorganism-associated molecular patterns
MAPK	mitogen-activated protein kinase
MetS	metabolic syndrome
mTOR	mammalian target of rapamycin
NAFL	non-alcoholic fatty liver
NAFLD	non-alcoholic fatty liver disease
NASH	non-alcoholic steatohepatitis
NF-kB	nuclear factor-κ B
OR	odds ratio
PGE2	Prostaglandin E2
RR	relative risk
SIR	standardized incidence ratio
STAT3	signal transducer and activator of transcription
TNF-α	tumor necrosis factor α
US	ultrasound
JAK2	Janus kinase-2
